# The Effects of Type 2 Diabetes on Cognitive Performance: A Review of Reviews

**DOI:** 10.1007/s12529-024-10274-6

**Published:** 2024-03-11

**Authors:** Teppo Sola, Fanny-Maria Sola, Mervi Jehkonen

**Affiliations:** 1 Psychology, Tampere University, Tampere, Finland; 2 Tampere University Hospital, Tampere, Finland

**Keywords:** Cognition, Cognitive performance, Diabetes, Neuropsychology, Review of reviews, Type 2 diabetes

## Abstract

**Background:**

Multiple systematic reviews have found that type 2 diabetes is associated with cognitive decrements. However, these reviews are heterogeneous in terms of methodology, quality and results, making it difficult for researchers and clinicians to build an informed overall picture. We therefore conducted a review of systematic reviews on the association between type 2 diabetes and cognitive decrements in relation to healthy controls.

**Methods:**

Following a pre-registered research protocol, we searched four major databases. Nine systematic reviews met our inclusion criteria: seven were meta-analyses and two were narrative syntheses. We assessed the risk of bias in each review and reported all effect sizes and confidence intervals obtained.

**Results:**

Type 2 diabetes was associated with cognitive decrements in all reviews, with small or negligible effect sizes obtained in the largest meta-analyses. The most studied cognitive domains were attention, executive functions, memory, processing speed and working memory. All reviews had methodological issues and were rated as having a high or an unclear risk of bias.

**Conclusions:**

Type 2 diabetes appears to be associated with lower cognitive performance in several cognitive domains and in different age groups. However, high-quality meta-analyses on the subject are still needed. Future reviews must follow the PRISMA guidelines and take into account the risk of bias of the original studies through sensitivity analyses and the heterogeneity of the studies by conducting subgroup analyses for example according to age group and disease duration. The meta-analyses that aim to study the entire type 2 diabetes population without excluding severe comorbidities, should assess concept formation and reasoning, construction and motor performance, perception, and verbal functions and language skills in addition to the cognitive domains that have been most frequently analysed in the reviews conducted so far.

**Supplementary Information:**

The online version contains supplementary material available at 10.1007/s12529-024-10274-6.

## Introduction

The association between diabetes and cognitive decrements was first reported as early as 1922, when the cognitive performance of diabetes patients was compared to healthy controls using neuropsychological tests [1]. In 1950, the term diabetic encephalopathy was coined to describe the central nervous system related complications of diabetes [2]. Since then it has been established that in older age, type 2 diabetes (T2DM) is a significant risk factor for the development of vascular dementia and Alzheimer’s disease, with risk ratios around 2.3 and 1.6, respectively [3]. However, longitudinal studies have demonstrated that T2DM patients can have mild diabetes-associated cognitive decrements that do not necessarily increase over time [4] or increase very slowly [5]. This is in line with results from brain imaging studies, which have found that although T2DM patients have slightly more global brain atrophy and vascular lesions than healthy controls even in middle age, these brain changes develop slowly over the course of many years [6]. Mild diabetes-associated cognitive decrements, mild cognitive impairment (MCI) and dementia should therefore be regarded as different stages of T2DM-associated cognitive dysfunction [7]. Because of the heterogeneity of the disease, the mechanisms behind the cognitive changes can differ greatly between individuals [7]. These mechanisms include, but are not limited to, microvascular tissue damage and advanced glycation end products caused by chronic hyperglycemia [8], decreased insulin-facilitated neural activity [9] and chronic inflammation, which has been associated with Alzheimer’s disease, vascular dementia and cognitive decline in older age [8].

In addition to longitudinal studies, there are dozens of cross-sectional studies that have compared the cognitive performance of T2DM patients and healthy controls using neuropsychological tests. The systematic reviews and meta-analyses of T2DM-associated cognitive decrements have mostly reported negligible-to-medium effect sizes in several cognitive domains [10–18]. However, the systematic reviews have used an array of different neuropsychological tests, classified cognitive domains in different ways or focused only on a certain age group or domain. The findings, too, have been somewhat heterogeneous: for example, some have reported a deterioration in overall memory functioning [12], while others have not [10]. In addition, some of the reviews have methodological problems that make their findings less robust or reliable. All this combines to make it difficult for clinicians or researchers to build an informed overall picture of diabetes-associated cognitive decrements.

Our review of systematic reviews seeks to address the aforementioned issues. We identify all systematic reviews that compare the cognitive performance of T2DM patients to that of healthy controls and report all of their results using the classification of cognitive domains by Lezak et al. [19]. We evaluate the risk of bias in the reviews and based on these evaluations offer our recommendations on the factors that should be taken into account in systematic reviews of T2DM-associated cognitive decrements. In addition, we report the most frequently analysed neuropsychological tests and discuss which cognitive domains should be studied further. To our knowledge, this is the first review of systematic reviews on cognitive performance in T2DM.

## Method

### Systematic Search

Preferred Reporting Items for Systematic Reviews and Meta-analysis (PRISMA) guidelines were followed throughout the review and the 27-item checklist was used [20]. An information specialist at Tampere University with expertise in systematic reviews was consulted to help develop the search strategy. The full search strategy is outlined in the pre-registered protocol (PROSPERO: CRD42021286148). We sought to identify all articles concerning type 1 or type 2 diabetes-associated cognitive decrements. Due to the large scope of the study and the different nature of the conditions, type 1 diabetes will be discussed in a separate article. We searched the following databases: Epistemonikos, PsycINFO, PubMed and Cochrane Library. All databases were first searched from inception until 1 November 2021. The search was re-executed on 12 October 2022 to include systematic reviews published after the initial search. No search restrictions were applied. Search terms concerning type 1 diabetes, type 2 diabetes and cognitive domains were used and combined with boolean operators OR and AND. In PubMed and PsycINFO we used additional search terms concerning systematic reviews and meta-analyses. Duplicates were removed using Zotero software version 5.0.96.3 [21]. Two reviewers (T.S. and F.S.) screened titles and abstracts independently against the inclusion criteria and disagreements were resolved through discussion. Two reviewers (T.S. and F.S.) also read the full texts and hand-searched reference lists independently against the inclusion criteria. Disagreements were resolved through discussion.

### Inclusion Criteria

We included all systematic reviews that (1) compared the cognitive performance of type 1 or type 2 diabetes patients and healthy controls, (2) reported quantitative data for at least one neuropsychological test or cognitive domain and (3) did not include studies with a primary focus on dementia, mild cognitive impairment (MCI) or any other neurological or psychiatric disorder unless comparisons were also available between diabetes patients without these conditions and healthy controls. We did not exclude studies based on language or diagnostic criteria used. Our protocol stated that we intend to include “systematic reviews of observational studies”. However, such a restriction would have led to relevant studies being unnecessarily excluded, so we decided to also include all systematic reviews that used the baseline data from randomized controlled trials.

### Risk of Bias Assessment

Two reviewers (T.S. and F.S.) assessed the risk of bias independently using ROBIS, an evidence-based rigorously developed tool for assessing risk of bias in systematic reviews [22]. Disagreements were resolved through discussion.

### Data Extraction

One reviewer (T.S.) extracted pre-specified data using Microsoft Excel and another reviewer (F.S.) checked that all data were extracted correctly. We extracted the following data about systematic reviews: title, authors, year of publication, type and source of funding, conflict of interests, objectives, risk of bias tool used, the results of risk of bias assessments, synthesis methods, search strategies, date ranges of searches, date of last search update, inclusion/exclusion criteria, number of primary studies and participants included, participant characteristics including age, sex, ethnicity and education, diagnostic criteria used to diagnose diabetes, duration of diabetes, HbA1c levels, treatment, comorbidities, names of the cognitive domains assessed, names of the neuropsychological tests used and all quantitative data concerning cognitive performance. We extracted basic information about primary studies, including authors, year of publication, study design and country of publication. The authors of the systematic reviews were contacted when insufficient data were reported.

### Synthesis

In this review of reviews, the unit of analysis is a systematic review rather than an individual study. We provide narrative summaries of neuropsychological test performance in patient and control groups, reporting effect sizes and 95% confidence intervals when possible. According to Cohen [23], an effect size of 0.2 is considered small, an effect size of 0.5 is medium and an effect size of 0.8 is large. When the effect size obtained is lower than 0.2 we call it negligible. An effect size is deemed statistically significant when the confidence interval for the effect size, typically at a 95% confidence level, does not include zero. This suggests there is a difference between the groups. It is important to note that a large sample size may result in statistically significant differences even with a negligible effect size, and vice versa. When interpreting the effect size, considering the practical significance of the findings is crucial.

Cognitive domains are not uniformly classified in different systematic reviews, and therefore the same neuropsychological test could belong to a different domain in different reviews. We categorize cognitive tests in the following domains, based on the widely used classification by Lezak et al. [19]: (1) attention, (2) concept formation and reasoning, (3) construction and motor performance, (4) executive functions, (5) memory, (6) perception, (7) processing speed, (8) verbal functions and language skills and (9) working memory, and report the results based on this categorization. Lezak et al. argue against using a composite score for intelligence [19], but we also report the results for intelligence/global cognition so as to not omit any results from the meta-analyses.

#### Overlap of Primary Studies

When reporting the results of several systematic reviews, it is important to note that the same primary study may have been included in more than one review. To avoid bias caused by such overlap, we used the Corrected Covered Area (CCA) approach [24]. In this method, primary studies and systematic reviews are recorded in a matrix. The frequency of repeated occurrences of an index primary study is divided by the product of index studies and reviews, and then multiplied by 100 to obtain the percentage of overlap. We applied the interpretation scheme where 0–5% overlap is considered a slight, 6–10% a moderate, 11–15% a high and over 15% a very high overlap of primary studies [24].

## Results

### Systematic Search Results

**Fig. 1:**
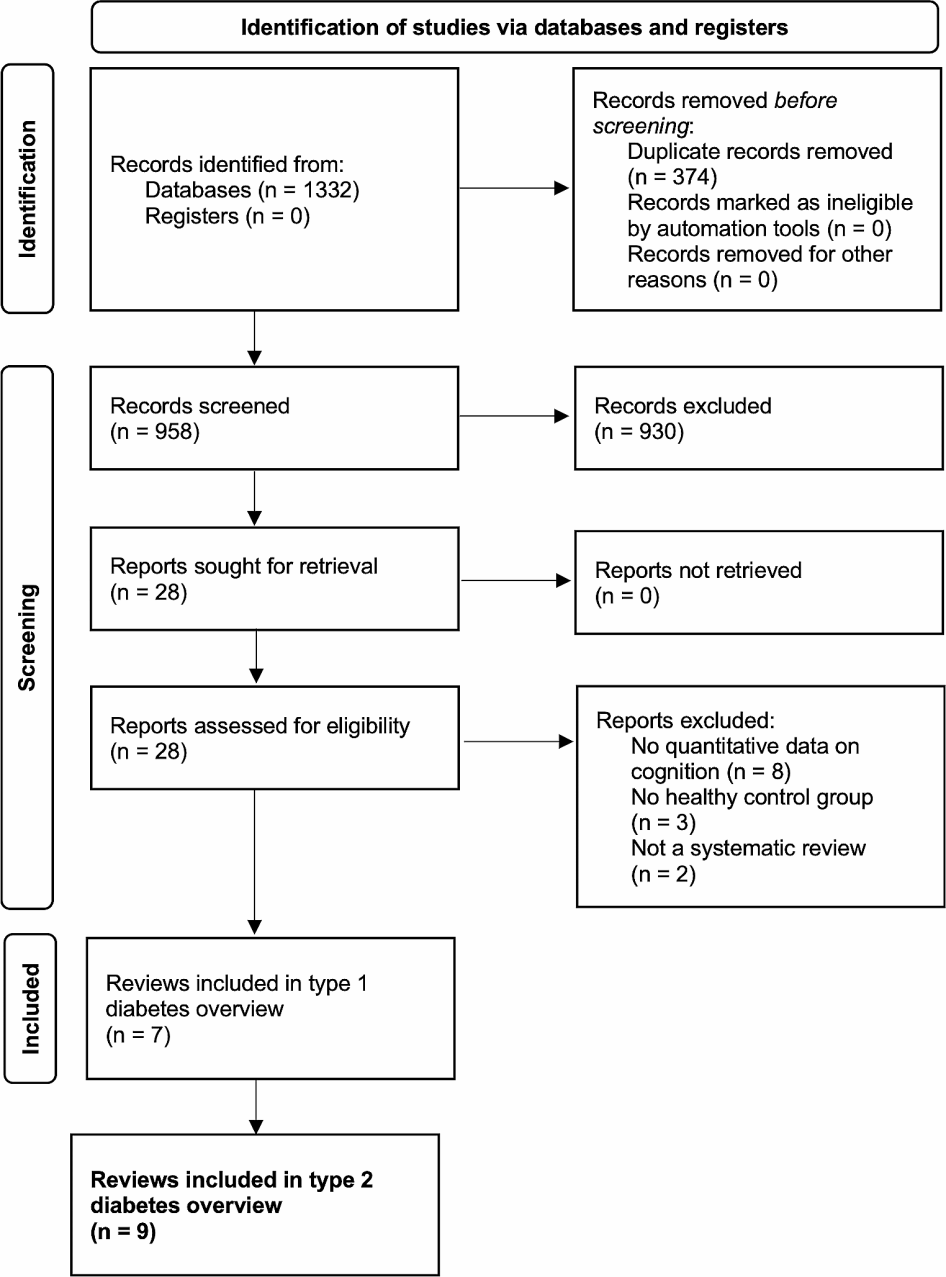
PRISMA Flowchart

The PRISMA flowchart in Fig. 1 outlines the search and the screening process. The initial search yielded 1156 results and the re-executed search 176 new results. After duplicates were removed, 958 articles remained for title and abstract screening and 28 articles were retrieved for full-text reading. No additional articles were found by searching reference lists. The reasons for exclusion of full-text articles were lack of quantitative data on cognition [25–32] and lack of a healthy control group [33–35]. Furthermore, two articles were not systematic reviews [36, 37]. After applying the inclusion criteria, a total of 15 articles remained. Six of the reviews were exclusively concerned with type 1 diabetes, eight exclusively with T2DM, and one review concerned both types of diabetes. Therefore, a total of nine T2DM reviews were included in this review. There was almost perfect agreement among the reviewers after title and abstract screening (Cohen’s kappa *k* = 0.90) and perfect agreement after full-text screening (*k* = 1.0).

### Risk of Bias

There were possible sources of bias in every systematic review included in this review. None of the nine reviews had a pre-registered protocol or included studies published in languages other than English. Most of the reviews either did not assess the risk of bias in the primary studies [10, 12, 17, 18] or did not address the recognized biases in the synthesis [10, 12, 15–18]. Most reviews did not use more than one author in the selection of studies [10–12, 14, 16] or in the data extraction process [10, 11, 13–15]. The other most common sources of biases were the use of insufficient search terms [10, 11, 14, 16, 17] or inappropriate range of databases or other electronic sources searched [10, 12, 14, 17]. The results of the risk of bias assessment are shown in Table 1.

**Table 1:** Risk of bias in systematic reviews

First author, year	Study eligibility criteria	Identification and selection of studies	Data collection and study appraisal	Synthesis and findings	Risk of bias
Kálcza-Jánosi [10]	-	-	-	-	-
Mansur [11]	+	-	?	+	-
Monette [12]	+	-	-	-	-
Palta [13]	+	+	?	+	?
Papunen [14]	+	-	-	+	-
Pelimanni [15]	+	-	-	+	-
Sadanand [16]	+	-	-	-	-
van den Berg [17]	+	-	-	-	-
Vincent [18]	+	-	-	+	-

Note + = low risk; - = high risk;? = unclear risk

Out of the seven meta-analyses reviewed in this study, four investigated publication bias [11, 12, 15, 18], and in three of them, bias was observed for at least one measure [11, 15, 18]. In two reviews, the identified publication bias was not accounted for in the analyses [11, 18], while one review addressed publication bias by removing outliers [15]. Three reviews did not address publication bias at all [10, 13, 16]. In all meta-analyses that assessed heterogeneity among original studies, it was observed in at least one cognitive domain or individual test performance [11–13, 15, 16, 18]. In most cases, efforts were made to correct for this heterogeneity using statistical methods.

### Study Characteristics

Table 2 describes the characteristics of the nine systematic reviews on T2DM and cognitive performance. Six reviews reported gender distributions, and in all of them the majority of patients and controls were women. Eight reviews reported age distributions, with the mean age of patients ranging from 57 to 72 years. Five reviews did not report HbA1c levels. In four reviews, mean HbA1c % value ranged between seven and eight. Mean duration of diabetes was reported in six reviews and it ranged from eight to 11 years.

**Table 2:** Characteristics of systematic reviews on T2DM and cognitive performance

First author (ref)	Synthesis method	k	*n* (patients/controls)	Males (%)	Age (M)	HbA1c % (M)	Duration in years (M)	Disease related exclusion criteria
Kálcza-Jánosi [10]	MA	6	215/221	NR	57.44/57.38 *R* = 51–62/51–62	8.45 *R* = 6.9–10.2 (k = 4, *n* = 144)	7.63 *R* = 6–9 (k = 5, *n* = 177)	not normal blood glucose values
Mansur [11]	MA	40	4252/22,322	NR	69.03/69.54 *R* = 46–84/48–83 (k = 38, *n* = 4172/22,161)	7.44 *R* = 5.7–13.3 (k = 28, *n* = 2260)	10.52 *R* = 4.7–15.8 (k = 25, *n* = 2403)	NR
Monette [12]	MA	25	1908/10,132	43.00/39.00 (k = 23, *n* = 1602/9663)	68.70/70.00 *R* = 48–85/48–84 (k = 23, *n* = 1676/8119)	7.55 *R* = 6.6–10.2 (k = 13, *n* = 767)	9.29 *R* = 4.7–13.8 (k = 13, *n* = 797)	hypo- or hyperglycemia induced by the study, diagnosed dementia
Palta [13]	MA	24	3351/22,786	23.40/4.75 (k = 21, *n* = 3110/22,165)	71.26/73.88 *R* = 51–85/51–85 (k = 22, *n* = 3145/20,670)	NR	NR	focus on acute alterations in blood glucose
Papunen [14]	NS	12	4983/34,818	NR	NR; participants > 60	NR	NR	MCI or dementia at baseline
Pelimanni [15]	MA	12	2139/12,287	43.72/44.86 (k = 8, *n* = 1987/12,157)	57.80/56.90 *R* = 48–63/45–63 (k = 10, *n* = 2069/12,237)	8.01 *R* = 6.7–10.2 (k = 8, *n* = 1981)	7.80 *R* = 6–8.5 (k = 6, *n* = 183)	focus on acute hypo- or hyperglycemia
Sadanand [16]	MA	15	2370/21,426	15.65/3.30	72.04/73.96 *R* = 56–84/54–83 (k = 14, *n* = 2191/19,442)	NR	11.42 *R* = 4.67–13.8 (k = 9, *n* = 1787)	MCI or dementia at baseline
van den Berg [17]	NS	27	5592/47,797	36.71 (matched) (k = 25, *n* = 5225/45,689)	66.19 (matched) *R* = 47–85 all (k = 24, *n* = 27,153 all)	NR	NR	none; exclusion criteria in included studies were reported
Vincent [18]	MA	60	9785/69,254	40.06/27.32 (k = 54, *n* = 8936/66,444)	69.63/69.13 *R* = 46–85/37–84 (k = 54, *n* = 9343/65,483)	NR	11.44 *R* = 0–14.6 (k = 25, *n* = 4868)	NR

Note All values are weighted. k = primary studies comparing patients and controls, MA = meta-analysis, NS = narrative synthesis, NR = not reported for one or both groups, R = range of means

### Results from the Meta-Analyses

Seven meta-analytic reviews investigated cognitive performance in T2DM. Two of them included studies covering all cognitive domains [10, 15], while the other five studied particular domains. The most commonly studied domains were attention, executive functions, processing speed, memory and working memory. The neuropsychological tests most frequently analysed were Trail Making Test B (7 reviews), Trail Making Test A (6), Digit Span Forward (6), Digit Span Backward (6), Digit Symbol Substitution Test (6), Rey Auditory Verbal Learning Test (5), Wechsler Intelligence Scale: Logical Memory (5), Stroop (5), Wisconsin Card Sorting Test (5), Phonemic Fluency (4), Semantic Fluency (4), California Verbal Learning Test (3) and Letter-number Sequencing (3). Other neuropsychological measures were analysed in fewer than three reviews.

When the performance in a single neuropsychological test was investigated in the meta-analysis, we classified the test according to Lezak et al. [19]. However, different reviews classified some of the same tests into different cognitive domains, which must be kept in mind when interpreting overall performance in cognitive domains. Specifically, the Trail Making Test A, Trail Making Test B, Stroop and Digit Symbol Substitution test were categorized under attention, executive functions or processing speed domains depending on the review.

Table 3 presents the results from the meta-analyses of overall performance in the cognitive domains as described by Lezak et al. [19]. The results for the cognitive subdomains and individual tests used are presented in Supplementary Material.

**Table 3:** Results of the meta-analyses of overall performance in cognitive domains

Cognitive domain	Review	k	*n* (gr 1 / gr 2)	SMD (95% CI)
Attention	Kálcza-Jánosi [10]	4	136/136	d = -0.33 (-0.56, -0.15)*
	Monette [12]	16	1440/7665	d = -0.29 (-0.34, -0.24)*
	Palta [13]	14	2418/20,725	d = -0.19 (-0.26, -0.12)*
	Pelimanni [15]	5	158/147	g = -0.55 (-0.80, -0.30)*
	Vincent [18]	27	25 669 total	d = -0.38 (-0.48, -0.29)*
Concept formation and reasoning^a^	-	-	-	-
Construction and motor performance^a^	-	-	-	-
Executive functions	Kálcza-Jánosi [10]	4	136/136	d = -0.32 (-0.21, -0.46)*
	Palta [13]	12	680/1104	d = -0.33 (-0.42, -0.24)*
	Pelimanni [15]	9	2001/12 173	g = -0.51 (-0.69, -0.34)*
	Vincent [18]	60	79 069 total	d = -0.25 (-0.30, -0.20)*
Memory	Kálcza-Jánosi [10]	4	141/141	d = -0.50 (-0.82, 0.07)
	Monette [12]	20	1410/8056	d = -0.20 (-0.28, -0.12)*
Perception^a^	-	-	-	-
Processing speed	Monette [12]	22	1678/7822	d = -0.33 (-0.38, -0.29)*
	Palta [13]	16	1381/1695	d = -0.33 (-0.41, -0.26)*
	Pelimanni [15]	10	2063/11,832	g = -0.68 (-0.84, -0.52)*
Verbal functions	Kálcza-Jánosi [10]	2	68/68	d = -0.36 (-0.45, -0.27)*
	Pelimanni [15]	3	78/463	g = -0.26 (-0.51, -0.02)*
Working memory	Kálcza-Jánosi [10]	3	86/86	d = 0.04 (-0.47, 0.54)
	Monette [12]	11	1163/6582	d = -0.20 (-0.24, -0.15)*
	Pelimanni [15]	5	177/161	g = -0.51 (-0.79, -0.22)*
	Vincent [18]	28	28 118 total	d = -0.13 (-0.19, -0.06)*

Note gr 1 = patient group, gr 2 = control group, SMD = standardized mean difference

^a^ overall performance was not investigated in the reviews; see “results from the meta-analyses” section for information

* statistically significant (*p* <.05)

#### Attention

Attention and its subdomains were investigated in seven reviews that covered a total of 26 meta-analyses. Two reviews did not report which primary studies were included in some of the meta-analyses and the authors could not be reached [16] or the data was not available [18]. In the other five reviews, a total of 58 unique primary studies were analysed. There was high overlap between these five reviews (CCA = 13%), with 21 primary studies included in more than one review.

##### Overall Attention

was investigated in five meta-analyses. Patients performed worse than controls in all of them, with effect sizes being *small* or *negligible* [10, 12, 13, 18], with the exception of the meta-analysis of middle-aged people, in which a *medium* effect size was observed [15].

##### Attention Subdomains

Performance in tests belonging to four attentional subdomains, namely attentional capacity, complex attention, concentration and divided attention, was examined in four to six meta-analytic reviews per domain. These included a large review [18] that investigated all subdomains, analysing 11 to 22 studies and a minimum of 4011 participants per each domain. Only statistically significant, and mostly *small*, effect sizes were found in all reviews [11–13, 15, 16, 18]. The review on middle-aged people found a *medium* effect size for all the attention subdomains. These analyses included 38 to 128 patients in total for attentional capacity, concentration and divided attention domains, while the result for the complex attention domain was obtained by analysing six studies with a total of 1925 patients [15]. However, the high number of patients in the complex attention domain was largely explained by the inclusion of one large study [38] in which a medium effect size was found.

#### Executive Functions

Executive functions and its subdomains were investigated in six reviews containing 16 meta-analyses. A total of 79 unique primary studies were analysed. Overlap between the reviews was moderate (CCA = 8%), with 22 studies included in more than one review.

##### Overall Executive Functions

Three meta-analyses observed a *small* effect size for overall executive functions performance [10, 13, 18]. These included an extensive meta-analysis which combined attention, inhibition, mental flexibility, verbal fluency and working memory domains [18]. A *medium* effect size was observed in a relatively large meta-analysis on middle-aged people [15].

##### Executive Functions Subdomains

Vincent & Hall [18] examined inhibition, mental flexibility and verbal fluency separately, finding a *small* effect size for all of them, based on 13 to 31 studies and thousands of participants in each domain. A *small* effect size for verbal fluency was also observed in a relatively large (k = 17, *n* = 1540 patients) meta-analysis [12].

*Negligible or small* effect sizes were reported in most meta-analyses that studied phonemic fluency and semantic fluency separately [12, 16, 18]. These included large meta-analyses containing six to 21 studies and a minimum of 568 patients [12, 16, 18]. The meta-analyses on middle-aged people found a *non-significant small* effect for phonemic fluency (k = 4, *n* = 1861 patients) and a *non-significant medium* effect for semantic fluency (k = 2, *n* = 45 patients [15]. However, by far the largest original study included in the meta-analysis observed a small effect size for phonemic fluency [38].

#### Memory

Two meta-analyses containing a total of 21 unique primary studies combined verbal and visual immediate and delayed recall measures to study overall memory performance [10, 12]. Overlap between the reviews was high (CCA = 14%): three studies were included in both analyses.

##### Overall Memory

The significantly larger of the two meta-analyses observed a *small* effect size for overall memory performance and for immediate and delayed recall analysed separately [12]. A small meta-analysis found a *non-significant medium* effect size for overall memory performance [10].

##### Verbal Memory

Verbal memory was analysed in six reviews containing 21 meta-analyses. A total of 35 unique primary studies were analysed, with high overlap between the reviews (CCA = 16%): 19 studies were included in more than one review.

A *small* effect size was observed in both meta-analyses that examined overall verbal memory performance by combining immediate and delayed recall measures. One of the meta-analyses included more studies (k = 15, *n* = 1349 patients [13]) than the other (k = 4, *n* = 1873 patients [15]), which only analysed middle-aged people. The high number of patients in the latter meta-analysis is largely explained by a major study in which only delayed recall was examined [38].

*Small-to-medium* effect sizes were observed for immediate recall of word-lists in three large meta-analyses that included nine to 13 studies and 658 to 2108 patients [11, 12, 16]. One review examined performance in California Verbal Learning Test (k = 2, *n* = 202 patients) and Rey Auditory Verbal Learning Test (k = 7, *n* = 891 patients) separately, finding a *small* effect for the former and a *non-significant negligible* effect for the latter. All four meta-analyses that studied delayed recall of word-lists observed a *small* effect [12, 13, CVLT; 13, RAVLT; 16]. These included two large meta-analyses that analysed 12 studies with 709 [12] and 2129 patients [16].

Immediate and delayed recall of stories were examined in four meta-analytic reviews. The largest meta-analysis found a *negligible* effect size for both conditions (k = 10, *n* = 748 patients in each), combining scores from four different memory tests [12]. The other three, significantly smaller meta-analyses with a maximum of four studies and 289 patients, observed *non-significant and mostly negligible* effect sizes, analysing only Wechsler Memory Scale: Logical Memory performance [13, 15, 16].

##### Visual Memory

Visual memory was studied in four reviews containing 11 meta-analyses. A total of 13 unique primary studies were analysed. Overlap between the reviews was moderate (CCA = 10%), with three of the studies being included in more than one review.

A *small* effect size was found in the largest of the two meta-analyses that studied overall visual memory performance (k = 6, *n* = 616 patients [13]). A small meta-analysis on middle-aged people found a *non-significant negligible* effect (k = 3, *n* = 88 patients [15]).

*Small* effect sizes were observed for immediate and delayed visual recall in the meta-analyses that contained four to five studies and a minimum of 250 patients each [12; 13, ROCF). However, one relatively large meta-analysis found *non-significant negligible* effect sizes for the immediate and delayed recall of Wechsler Memory Scale: Visual reproduction performance based on two studies with 208 patients in each condition [13]. A small meta-analysis with only two studies and 88 patients observed *negligible* effect sizes for immediate and delayed visual recall [10], while a small meta-analysis on middle-aged patients found a *medium* effect for delayed visual recall (k = 2, *n* = 38 patients [15]).

#### Working Memory

Working memory was investigated in six reviews containing eight meta-analyses. A total of 39 unique primary studies were analysed. Overlap between the reviews was high (CCA = 11%), with 16 studies being included in more than one review.

##### Overall Working Memory

A meta-analysis that was clearly larger than the others found a *negligible* effect size for overall working memory performance [18]. A smaller but still relatively large meta-analysis observed a *small* effect [12], while the relatively small meta-analysis on middle-aged people found a *medium* effect [15].

##### Working Memory Subdomains

Performance in Digit Span Backward was analysed separately in four meta-analyses. By far the largest of them (k = 18, *n* = 26 992 participants) found a *small* effect [18], while a *negligible* effect was observed in two other meta-analyses that contained eight to nine studies [13, 16]. A small meta-analysis (k = 3, *n* = 95 patients) on middle-aged people observed a *non-significant medium* effect [15].

#### Processing Speed

Processing speed was investigated in five reviews that contained a total of 13 meta-analyses. One review did not report which primary studies were analysed and the data was not available [18]. The other four reviews contained a total of 50 unique primary studies. Overlap between these reviews was high (CCA = 13%), with 17 studies being included in more than one review.

##### Overall Processing Speed

Three meta-analyses investigated overall processing speed performance. In each of them patients performed significantly worse than controls. A *small* effect size was observed in the largest two meta-analyses [12, 13], while the relatively large meta-analysis on middle-aged patients found a *medium* effect. In one review, processing speed tasks with motor task demands (k = 21, *n* = 1551 patients) and oral task demands (k = 6 *n* = 388 patients) were analysed separately, and *a small and a negligible* effect size were observed, respectively [12].

##### Processing Speed Subdomains

Four reviews comprising a total of eight meta-analyses analysed performance on a single processing speed test. *Small* effect sizes were found for reading and colour naming conditions of Stroop (k = 6, *n* = 516 patients [13]). Three large meta-analyses with 11 to 22 studies and a minimum of 811 patients in each observed a *small* effect for Trail Making Test A [11, 13, 18]. In the meta-analysis on middle-aged people a *medium* effect was found for Trail Making Test A based on three studies with a total sample of only 53 patients [15]. They also observed a *medium* effect for Choice Reaction Time and a *small* non-significant effect for Simple Reaction Time, with both of these analyses including two studies and only 66 patients.

#### Perception

Perception as defined by Lezak et al. [19] was not analysed as a separate domain in any of the meta-analyses. Two meta-analyses examined perception/construction. There were 11 unique primary studies and overlap between the reviews was moderate (CCA = 9%), with one study included in more than one review.

The larger of the two meta-analyses (k = 7, *n* = 493 patients) observed a *negligible* effect [12], while the smaller meta-analysis on middle-aged people (k = 6, *n* = 158 patients) found a *small* effect for overall perception/construction performance [15]. The smaller meta-analysis [15] also analysed “ROCF: copying score” separately and observed a *medium* effect size (k = 3, *n* = 70 patients).

#### Concept Formation and Reasoning

None of the meta-analyses examined concept formation and reasoning. The neuropsychological tests categorized under this domain by Lezak et al. [19] were analysed as part of other cognitive domains in some of the meta-analyses. Palta et al. [13] found a *non-significant small* effect of d = -0.35, 95% CI [-0.70, 0.00] for Wisconsin Card Sorting Test (categories) based on two studies that included a total of only 48 patients.

#### Construction and Motor Performance

Monette et al. [12] analysed motor speed and observed a *small* effect size (k = 4, *n* = 360 patients), while Palta et al. [13] found a *small* effect size for motor function (k = 3, *n* = 294 patients). Palta et al. [13] also analysed Grooved Pegboard Test performance for dominant and non-dominant hand separately, finding *medium* effect sizes (k = 2, *n* = 115 patients) in both conditions.

#### Verbal Functions and Language Skills

Verbal functions and language skills were examined in two small meta-analyses. A total of five unique primary studies were included and there was no overlap between the reviews (CCA = 0%). One review included two studies with a total of 68 patients [10] and the other included three studies with a total sample of 78 middle-aged patients [15]. A *small* effect size was found in both meta-analyses.

#### Intelligence / Global Cognition

A large meta-analysis that included 25 studies and 1908 patients combined all neuropsychological tests from several different cognitive domains and found a *small* effect size for global cognition [12]. They also analysed non-verbal reasoning, identifying six studies with a total sample of 333 patients. Again, a *small* effect size was observed. One meta-analysis [10] observed a *medium* effect size for intelligence, based on three studies with a total sample of 109 patients.

### Results from the Narrative Syntheses

Two narrative syntheses investigated cognitive performance in T2DM, both of which included studies on all cognitive domains [14, 17].

One narrative synthesis found that although 11 of the 17 original studies reported a statistically significant decline in cognition among older T2DM or pre-diabetes patients, the association was not always clear-cut and the effects were largely explained by the extent of neuropsychological tests used in an individual study [14]. Significant associations were found more often when a global cognition measure was used instead of a test that focuses on a specific domain. Another narrative synthesis, which included 27 studies on T2DM and cognitive performance, found statistically significant worsening in 13 out of 20 cross-sectional and in five out of seven longitudinal studies [17]. Processing speed was affected in 63% of the studies assessing that domain and attention in 50% of the studies, with median effect sizes of -0.4 observed for the former and − 0.5 for the latter. Memory, cognitive flexibility, language, general intelligence, and perception and construction were affected in fewer than half of the studies assessing these domains. The cross-sectional studies in relatively older patients obtained larger effect sizes than those in younger patients, and the results were similar in the six studies that adjusted for vascular risk factors.

## Discussion

The aim of our review was to identify all systematic reviews that examined cognitive performance of individuals with T2DM compared to healthy controls, evaluate the risk of bias in these reviews, report their findings, and identify the most frequently used tests as well as lesser studied cognitive domains. We found two narrative syntheses and seven meta-analyses. In the meta-analyses the number of patients ranged from a few hundred to thousands.

In the meta-analyses, the most commonly studied cognitive domains were attention, executive functions, processing speed, memory, and working memory. The most analysed neuropsychological tests were mainly those widely used in research and clinical practice, such as Trail Making Test, Stroop and Rey Auditory Verbal Learning Test. However, when looking at the overall performance in cognitive domains, it must be taken into account that different meta-analyses classified some of the same tests as belonging to different domains. This might lead different reviews referring to different cognitive domains despite observing impairments within the same domains in reality. Given that most neuropsychological tests are known to measure multiple cognitive processes simultaneously, the challenge of classifying tests is a recognized issue in most studies investigating cognitive performance.

In all meta-analyses, patients performed worse than healthy controls in at least one cognitive domain. Furthermore, in the meta-analyses that examined performance on individual neuropsychological tests, patients generally performed worse than controls. In the large meta-analyses, which included patients from all age groups, effect sizes for the attention and working memory domains ranged between negligible and small. For executive functions, memory, and processing speed, the effect sizes were mostly small. Overall, in these large meta-analyses, effect sizes across cognitive domains ranged between d = -0.13 and d = -0.38. In the only meta-analysis where the risk of bias was assessed as uncertain rather than high, the effects were of similar size [13].

It is interesting that the larger of the two meta-analyses limited to middle-aged patients consistently found bigger effect sizes for the cognitive domains than the other meta-analyses, with most effect sizes being medium. The only exception was visual memory, in which no group difference was found [15]. The authors discuss the surprisingly large effect sizes and consider possible explanations. These include the small number of relevant studies in certain cognitive domains in their review, the publication bias observed, insufficient control of confounding in some primary studies, differences in how the reviews categorize neuropsychological tests, and the fact that most of the studies in their review were case-control studies, in which effect sizes are often larger than in population-based and longitudinal studies. However, they also considered the possibility that there is a stronger association between diabetes and cognitive decrements in younger age. They argue that this could be due to the fact that ageing and increased morbidity also impair the cognitive performance of older controls who do not have diabetes [39–40, as cited by 15], and the cognitive changes observed in T2DM could develop during a pre-diabetes stage and remain relatively stable over time. In our own recent study, that used strict disease-related exclusion criteria and included 28 middle-aged T2DM patients and 28 age-, education- and gender-matched healthy individuals, we did not find between-group differences in any of the 21 neuropsychological outcome measures analysed [41]. The effect sizes in our study were non-significant and mostly negligible or small, with the mean effect size being − 0.12. Furthermore, the other, smaller, meta-analysis that only included middle-aged patients as well, reported small effect sizes for some of the same domains that were analysed in the other meta-analysis [15], namely attention and executive functions [10]. We believe that the heterogeneity and methodological issues of the studies included in the meta-analysis by Pelimanni & Jehkonen [15] explains the larger effect sizes obtained.

In the meta-analyses, the less investigated cognitive domains were concept formation and reasoning, construction and motor performance, perception, and verbal functions and language skills, although the meta-analyses that analysed tests from these domains reported mostly small or negligible effect sizes [10, 12, 13, 15]. The focus on researching attention, executive functions, processing speed, and memory functions is understandable because of the well-established understanding that performance in these cognitive domains typically declines more in cerebral small vessel disease compared to, for example, language functions or perception [e.g. 42, 43]. Cerebral small vessel disease is the most common pathology associated with vascular cognitive impairment and vascular changes are considered one of the primary mechanisms behind the cognitive symptoms observed in T2DM [e.g. 8]. Additionally, brain imaging studies in individuals with T2DM have revealed general brain atrophy and vascular lesions that seem to progress slowly over several years [6]. It is not plausible that T2DM without severe comorbidities causes specific neuropsychological disorders typically associated with stroke or dementia, such as agnosia, neglect or aphasia, because these disorders require severe damage to specific brain regions. It is also of note that even though the meta-analyses indicated impaired memory performance, the effect sizes for immediate and delayed recall measures were of similar size, and none of the meta-analyses investigated the extent of forgotten material after a delay from the initial learning phase. Hence, it is reasonable to consider that the worse memory performance in patients could be explained by the attentional, processing speed, and executive requirements of the memory tests, rather than being ascribed to the delayed memory loss commonly observed in conditions such as Alzheimer’s disease but not in vascular cognitive impairment.

Since the cognitive symptoms of people with T2DM can vary from mild subjective symptoms to dementia, it is not completely clear whether the differences observed between groups in the meta-analyses are due to significant proportion of the diabetes patients performing slightly worse than the healthy controls or a smaller subset of patients performing significantly worse. This is because in the majority of the meta-analyses, mild cognitive impairment, dementia, cerebrovascular disease, or psychiatric illness were not set as exclusion criteria. Furthermore, subgroup analyses focusing solely on studies that used certain diabetes complications or comorbidities as exclusion criteria were not performed. Therefore, there does not seem to be compelling meta-analytic evidence at this time that T2DM without complications or comorbidities causes cognitive symptoms.

In a narrative synthesis that focused on elderly patients (over 65 years of age at the start of the study) and excluded mild cognitive impairment or dementia at baseline, it was found that even though most of the original studies reported a significant decline in cognition among T2DM patients, the association was not always unambiguous, and the number of neuropsychological tests used seemed to have the greatest effect on the results [14]. A narrative synthesis by van den Berg et al. [17], which only included age-, gender-, and education-adjusted or -matched studies and also excluded studies containing individuals with dementia, reported a negative association between T2DM and cognitive performance. Processing speed and attention were found to be affected in most studies that assessed these domains with median effect sizes being small and medium, respectively. Higher age appeared to be associated with poorer performance.

It would be important to understand if the cognitive changes observed at the group level in the systematic reviews are reversible through optimally managed glucose levels. In the narrative syntheses, acute blood glucose fluctuations were not the focus of interest. Three of the meta-analyses did not include primary studies specifically focusing on acute hypo- or hyperglycemia [10, 13, 15]. Four meta-analyses either included studies addressing acute blood glucose alterations [12, 16] or did not specify disease-related inclusion criteria [11, 18]. However, the exclusion of studies concentrating solely on these aspects does not guarantee that some patients analysed in the primary studies did not experience acute hypo- or hyperglycemia. Acute hypoglycemic episodes are less common in T2DM than in type 1 diabetes, but they can occur if insulin or sulphonylurea medications are used [44]. Acute hypoglycaemia can temporarily impair reaction time, memory, attention, verbal fluency, executive function, and visuospatial abilities [e.g. 45], and at least in the older population, recurrent hypoglycemic episodes are associated with poorer cognitive performance [46]. Acute hyperglycaemia has also been found to temporarily impair processing speed, working memory and attention [47]. Determining whether the cognitive symptoms observed in the systematic reviews might be reversible would necessitate analyzing longitudinal or intervention studies. As mentioned before, it is possible that some patients analysed in the meta-analyses might have developed dementia, in which case their symptoms would not be reversible. However, it is reasonable to assume that a larger group of T2DM patients exhibit milder cognitive symptoms that could potentially be reversible by optimal management of the diabetes.

### Implications for Research

The results of the systematic reviews included in this review should be treated with caution since the risk of bias in all the reviews was considered to be high or unclear. We strongly recommend pre-registering the research protocol when conducting a systematic review. This makes the research process more transparent and reduces bias in the conduct and reporting of the research [48]. Searches and inclusion criteria should not be restricted to research published in the English language. This is common practice but will introduce language bias and increase the risk of overlooking valuable findings and missing important cultural contexts [49]. There are ways of including foreign language studies even without the need for expensive full translations of articles [49]. The selection of the studies and data extraction should be done by a minimum of two researches in order to avoid potentially significant mistakes in the process. The risk of bias in the primary studies included in the meta-analysis should be rated by a minimum of two authors and taken into account in the synthesis, for example by conducting a sensitivity analysis to omit studies with a high risk of bias. Multiple methods exist for assessing the risk of bias in observational studies [e.g. 50]. Some of the reviews failed to identify primary studies included in the other reviews, most likely because of insufficient search terms concerning cognitive domains or an insufficient number of databases searched. It is recommended to consult an information specialist with expertise in search methodology when designing the search strategy.

In addition to assessing the risk of bias of the original studies, the systematic review authors should pay attention to how the different factors through which T2DM can impair cognitive performance are considered and controlled in the original studies. These include, but are not necessarily limited to, anxiety, depression, dyslipidemia, fatigue, hypertension, hypothyroidism and obesity. The authors of the reviews must decide whether they are interested in the T2DM population as a whole, a subgroup of patients that do not have certain comorbidities, or in both. This is important because T2DM-associated cognitive decrements could be attributable to confounding variables if these are not identified as well as possible. On the other hand, if comorbidities typical for individuals with diabetes are used as exclusion criteria, the results of meta-analyses no longer correspond to the actual population of individuals with diabetes. In this case, the cognitive performance of individuals with T2DM is likely to be worse at the population level than suggested by such meta-analyses. In addition, it is clear that potential risk factors for developing diabetes, such as advanced age, low educational level and low socioeconomic status, are also risk factors for cognitive symptoms in the general population. Since matching or adjusting by age, education and sex was not set as an inclusion criterion in the meta-analyses, the group differences observed in some of the analyses might be due to patient groups’ lower premorbid cognitive ability. However, it has been reported that the magnitude of cognitive decrements associated with metabolic syndrome and other pre-diabetes stages are smaller than the decrements observed in T2DM [51].

In future meta-analyses, especially when they do not employ strict exclusion criteria regarding comorbidities associated with T2DM and therefore might include individuals with, for example, cerebrovascular disease or memory disorder, we hope to see more investigation into cognitive domains that have received less attention.These include concept formation and reasoning, construction and motor performance, everyday attention, perception, and verbal functions and language skills, as classified by Lezak et al. [19]. It would also be important to study cognitive performance in different age groups. To our knowledge, only two meta-analyses have investigated T2DM-associated cognitive decrements in middle-aged people, one of which identified 12, mostly relatively small primary studies [15] and the other of which identified six studies with 50 patients analysed in the largest of them [10]. Both of these reviews were assessed as having a high risk of bias.

### Implications for Behavioral Medicine

Patients, following diagnosis, primarily take charge of managing diabetes themselves, aiming for a normal lifespan, optimal quality of life, and prevention of additional health issues. It is reasonable to assume that more severe cognitive symptoms notably impact this ability. A review highlighted that poorer cognitive performance in T2DM patients over 55 years old correlated with factors such as limited diabetes knowledge, reduced frequency of self-care activities, and difficulties in managing insulin doses and adhering to medications [52]. However, several methodological challenges were discussed in the review. Heterogeneity across original studies prevented a meta-analysis, and most studies lacked a control group. Moreover, independent effects of aging on diabetes self-management were not adequately explored. The review identified a need for further research in this area.

In middle-age, cognitive symptoms associated with T2DM can be subjective or, at least when there are comorbidities, detected through neuropsychological testing [7]. Mild symptoms are believed to develop in the prediabetes stage and progress very slowly over the years, often without a single identifiable explanatory mechanism. These symptoms do not necessarily require closer monitoring unless, according to the patient, their family, or healthcare professionals, they impair daily functioning or diabetes self-management. In these situations or when mild cognitive impairment (MCI) or dementia is suspected, consultation with neurologist and, if necessary, neuropsychologist, can be sought. Sometimes symptoms may progress rapidly, necessitating close monitoring and a reassessment of treatment. The American Diabetes Association recommends screening for cognitive symptoms in all people with diabetes over the age of 65 [53].

In scientific studies, efforts have been made to prevent or alleviate cognitive symptoms associated with T2DM through lifestyle guidance aiming for good disease management and prevention of complications. In a Finnish study, patients receiving individualized guidance on diet, exercise, and weight management performed cognitively similarly nine years after the intervention compared to patients in the control group who received only general health advice [54]. In a review, interventions based on physical activity or exercise did not seem to improve cognition in patients with T2DM, insulin resistance, or impaired glucose tolerance [55]. The research evidence regarding the protective effect of medication on cognition is not consistent. In some studies, metformin and sulphonylureas have been associated with reduced dementia risk, while in other studies, metformin, insulin treatment, and glitazone have been linked to increased cognitive symptoms and dementia risk [56]. In a longitudinal study involving individuals over 70 years of age with comorbid Alzheimer’s disease and T2DM, slower cognitive decline was observed compared to patients who only had Alzheimer’s disease [57]. The medication aimed at reducing blood glucose levels was thought to have a protective effect on cognition.

Despite the lack of convincing evidence for the direct protective effect on cognition through personalized lifestyle guidance, exercise interventions, or medication, factors critical in preventing the development of cognitive symptoms in prediabetic stages or in slowing cognitive decline in T2DM can be discerned from research. The most predictive factors for dementia in T2DM patiens during a ten-year follow-up were microvascular disease, diabetic foot, cerebrovascular disease, cardiovascular disease, acute metabolic events, depression, age and education [58]. The dementia risk was approximately 5% for those with the lowest risk score and 73% for those with the highest risk score [58]. The key modifiable risk factors to prevent some of the more severe T2DM complications include at least blood pressure, dyslipidemia, obesity, and poor diabetes management. Lifestyle modifications can often help manage these risk factors and it is crucial for patients to receive personalized information about the significance of these factors in preventing diabetes complications and dementia. Additionally, timely attention to depression, anxiety, and fatique symptoms is essential, directing patients to necessary treatment when needed. Striving for tight glycemic control is no longer recommended in advanced stages of memory disorders, as hypoglycemia might exacerbate the progression and severity of cognitive symptoms [56].

Clinical neuropsychologists and other healthcare professionals working with people with diabetes should be aware that T2DM have been associated with cognitive symptoms and, based on research evidence, is also a risk for vascular dementia and Alzheimer’s disease at a later age [3]. Professionals who are aware of T2DM-associated decrements are probably less likely to associate certain subjective or objectively ascertainable symptoms with some other, less likely cause. In addition, recognizing the tests in which decrements have been observed in T2DM patients can potentially help the clinical neuropsychologist to choose appropriate tests when conducting a neuropsychological assessment.

#### Strengths and Limitations

This review of systematic reviews has some limitations. The systematic reviews included in our review used different categorizations to classify neuropsychological tests into cognitive domains. For example, Trail Making Test B was considered an attention measure in some reviews [12], and an executive functions measure in others [10, 11, 13, 15–18]. We used the classification provided by Lezak et al. [19] to group tests into cognitive domains. Some readers might argue that there are more appropriate systems.

It goes without saying that the results in this review will reflect the results obtained in the systematic reviews included. As discussed earlier, all of the reviews included here were assessed to have a high or unclear risk of bias, which is why the results presented in this review must be considered with caution. Furthermore, the overlap of primary studies included in more than one systematic review was high in most of the cognitive domains where group differences were observed. Therefore, the similarity of effect sizes across different meta-analyses could at least partly reflect the fact that they analysed some of the same studies. The possible biases in these studies could affect the results of multiple meta-analyses since most of them did not assess the risk of bias in original studies or consider it when conducting the synthesis. Furthermore, many reviews observed heterogeneity or publication bias for at least one neuropsychological measure, which further complicates the interpretation of the results. Even though in most reviews efforts were made to correct for heterogeneity using statistical methods, performing a subgroup analysis could often be a more fruitful approach. In this case, different meta-analyses would be performed depending on factors such as comorbidities, disease duration or age of the patients. However, this approach may not be feasible if there is not a sufficient number of methodologically high-quality original studies available.

Despite its limitations, this review of reviews has several strengths as well. The PRISMA guidelines for systematic reviews were followed throughout the study. We registered our research protocol in advance and rigorously followed it. We used a comprehensive search strategy and the search was re-executed at a later stage in order to include more recent reviews. We did not restrict our search based on language or other sources of information. The identification of studies, data extraction and the risk of bias assessment were done by two researchers independently, and all quantitative data from the meta-analyses were reported.

## Conclusions

T2DM was associated with cognitive decrements in all systematic reviews included in this review. The effect sizes observed in the largest meta-analyses were mostly small or negligible, with the most affected domains being attention, executive functions, memory, processing speed and working memory. The other domains, such as perception and language, have not been studied as extensively, but small meta-analyses have observed group differences with negligible to small effect sizes in these domains as well. All systematic reviews had methodological issues and were rated as having a high or unclear risk of bias. Therefore, high-quality meta-analyses on the subject are still needed.

To our knowledge, this is the first review of systematic reviews on T2DM-associated cognitive decrements. We believe the information from this review will help researchers to plan future studies and clinicians to identify the cognitive domains potentially affected by T2DM.

## Electronic Supplementary Material

Below is the link to the electronic supplementary material.

Supplementary Material 1

## Data Availability

There is no dataset associated with this review of reviews. All results from systematic reviews are presented in supplementary material.

## References

[1] Miles WR, Root HF. Psychologic tests applied to diabetic patients. Arch Intern Med (Chic). 1922;30(6):767–77.

[2] DeJong RN. The nervous system complications in diabetes mellitus with special reference to cerebrovascular changes. J Nerv Ment. 1950;111:181–206.

[3] Gudala K, Bansal D, Schifano F, Bhansali A. Diabetes mellitus and risk of dementia: a meta-analysis of prospective observational studies. J Diabetes Investig. 2013;4(6):640–50. 24843720 10.1111/jdi.12087 PMC4020261

[4] Bangen KJ, Gu Y, Gross AL, et al. Relationship between type 2 diabetes Mellitus and Cognitive Change in a multiethnic Elderly Cohort. J Am Geriatr Soc. 2015;63(6):1075–83. 26096383 10.1111/jgs.13441 PMC4477832

[5] Pappas C, Andel R, Infurna FJ, Seetharaman S. Glycated haemoglobin (HbA1c), diabetes and trajectories of change in episodic memory performance. J Epidemiol Community Health. 2017;71(2):115–20. 27440936 10.1136/jech-2016-207588

[6] Brundel M, Kappelle LJ, Biessels GJ. Brain imaging in type 2 diabetes. Eur Neuropsychopharmacol. 2014;24(12):1967–81. 24726582 10.1016/j.euroneuro.2014.01.023

[7] Biessels GJ, Despa F. Cognitive decline and dementia in diabetes mellitus: mechanisms and clinical implications. Nat Rev Endocrinol. 2018;14(10):591–604. 30022099 10.1038/s41574-018-0048-7 PMC6397437

[8] Mayeda ER, Whitmer RA, Yaffe K. Diabetes and cognition. Clin Geriatr Med. 2015;31(1):101–ix. 25453304 10.1016/j.cger.2014.08.021 PMC4370221

[9] Arnold SE, Arvanitakis Z, Macauley-Rambach SL, et al. Brain insulin resistance in type 2 diabetes and Alzheimer disease: concepts and conundrums. Nat Rev Neurol. 2018;14(3):168–81. 29377010 10.1038/nrneurol.2017.185 PMC6098968

[10] Kálcza-Jánosi K, Lukács A, Barkai L, Szamosközi I. Kognitív funkciók az 1-es és 2-es típusú cukorbetegségben. Metaanalízis [Cognitive functions in type 1 and type 2 diabetes. Meta-analysis]. Orv Hetil. 2013;154(18):694–9. 23628730 10.1556/OH.2013.29602

[11] Mansur RB, Lee Y, Zhou AJ, et al. Determinants of cognitive function in individuals with type 2 diabetes mellitus: a meta-analysis. Ann Clin Psychiatry. 2018;30(1):38–50. 29373617

[12] Monette MC, Baird A, Jackson DL. A meta-analysis of cognitive functioning in nondemented adults with type 2 diabetes mellitus. Can J Diabetes. 2014;38(6):401–8. 24933107 10.1016/j.jcjd.2014.01.014

[13] Palta P, Schneider AL, Biessels GJ, Touradji P, Hill-Briggs F. Magnitude of cognitive dysfunction in adults with type 2 diabetes: a meta-analysis of six cognitive domains and the most frequently reported neuropsychological tests within domains. J Int Neuropsychol Soc. 2014;20(3):278–91. 24555960 10.1017/S1355617713001483 PMC4132660

[14] Papunen S, Mustakallio-Könönen A, Auvinen J, Timonen M, Keinänen-Kiukaanniemi S, Sebert S. The association between diabetes and cognitive changes during aging. Scand J Prim Health Care. 2020;38(3):281–90. 32777967 10.1080/02813432.2020.1802140 PMC7470088

[15] Pelimanni E, Jehkonen M. Type 2 diabetes and cognitive functions in Middle Age: a Meta-analysis. J Int Neuropsychol Soc. 2019;25(2):215–29. 30575498 10.1017/S1355617718001042

[16] Sadanand S, Balachandar R, Bharath S. Memory and executive functions in persons with type 2 diabetes: a meta-analysis. Diabetes Metab Res Rev. 2016;32(2):132–42. 25963303 10.1002/dmrr.2664

[17] van den Berg E, Kloppenborg RP, Kessels RP, Kappelle LJ, Biessels GJ. Type 2 diabetes mellitus, hypertension, dyslipidemia and obesity: a systematic comparison of their impact on cognition. Biochim Biophys Acta. 2009;1792(5):470–81. 18848880 10.1016/j.bbadis.2008.09.004

[18] Vincent C, Hall PA. Executive function in adults with type 2 diabetes: a Meta-Analytic Review. Psychosom Med. 2015;77(6):631–42. 25469685 10.1097/PSY.0000000000000103

[19] Lezak MD, Howieson DB, Bigler ED, Tranel D. Neuropsychological assessment. 5th ed. New York, NY: Oxford University Press; 2012.

[20] Page MJ, McKenzie JE, Bossuyt PM, et al. The PRISMA 2020 statement: an updated guideline for reporting systematic reviews. BMJ. 2021;372:n71. Published 2021 Mar 29. 33782057 10.1136/bmj.n71 PMC8005924

[21] Roy Rosenzweig Center for History and New Media. Zotero [Computer software]. 2016. Retrieved from www.zotero.org/download.

[22] Whiting P, Savović J, Higgins JP, et al. ROBIS: a new tool to assess risk of bias in systematic reviews was developed. J Clin Epidemiol. 2016;69:225–34. 26092286 10.1016/j.jclinepi.2015.06.005 PMC4687950

[23] Cohen J. Statistical Power Analysis for the behavioral sciences. Taylor and Francis; 2013. Rev. ed.

[24] Pieper D, Antoine SL, Mathes T, Neugebauer EA, Eikermann M. Systematic review finds overlapping reviews were not mentioned in every other overview. J Clin Epidemiol. 2014;67(4):368–75. 24581293 10.1016/j.jclinepi.2013.11.007

[25] Arvanitakis Z, Wilson RS, Bennett DA. Diabetes mellitus, dementia, and cognitive function in older persons. J Nutr Health Aging. 2006;10(4):287–91. 16886098

[26] Coker LH, Shumaker SA. Type 2 diabetes mellitus and cognition: an understudied issue in women’s health. J Psychosom Res. 2003;54(2):129–39. 12573734 10.1016/s0022-3999(02)00523-8

[27] Dong Y, Kua ZJ, Khoo EY, Koo EH, Merchant RA. The utility of brief cognitive tests for patients with type 2 diabetes Mellitus: a systematic review. J Am Med Dir Assoc. 2016;17(10):889–95. 27461866 10.1016/j.jamda.2016.06.010

[28] Fernandes-Lopes RM, de Lima-Argimon II. Elderly people with diabetes mellitus type 2 and cognitive performance in the Wisconsin Card sorting test (WCST). Univ Psychol. 2010;9(3):697–713.

[29] Li W, Huang E, Gao S. Type 1 diabetes Mellitus and cognitive impairments: a systematic review. J Alzheimers Dis. 2017;57(1):29–36. 28222533 10.3233/JAD-161250

[30] Niermeyer MA. Cognitive and gait decrements among non-demented older adults with type 2 diabetes or hypertension: a systematic review. Clin Neuropsychol. 2018;32(7):1256–81. 29261088 10.1080/13854046.2017.1414306

[31] Podolski N, Brixius K, Predel HG, Brinkmann C. Effects of regular physical activity on the cognitive performance of type 2 Diabetic patients: a systematic review. Metab Syndr Relat Disord. 2017;15(10):481–93. 29160740 10.1089/met.2017.0120

[32] Zhang X, Jiang X, Han S, Liu Q, Zhou J. Type 2 diabetes Mellitus is Associated with the risk of cognitive impairment: a Meta-analysis. J Mol Neurosci. 2019;68(2):251–60. 30949957 10.1007/s12031-019-01290-3

[33] Rama Chandran S, Jacob P, Choudhary P. A systematic review of the effect of prior hypoglycaemia on cognitive function in type 1 diabetes. Ther Adv Endocrinol Metab. 2020;11:2042018820906017. Published 2020 Feb 14. 32110374 10.1177/2042018820906017 PMC7025428

[34] Chen YX, Liu ZR, Yu Y, Yao ES, Liu XH, Liu L. Effect of recurrent severe hypoglycemia on cognitive performance in adult patients with diabetes: a meta-analysis. J Huazhong Univ Sci Technolog Med Sci. 2017;37(5):642–8. 29058275 10.1007/s11596-017-1784-y

[35] Peñaherrera-Oviedo C, Moreno-Zambrano D, Palacios M, et al. Does intensive glucose control prevent Cognitive decline in diabetes? A Meta-analysis. Int J Chronic Dis. 2015;2015:680104. 26464871 10.1155/2015/680104 PMC4590930

[36] Koekkoek PS, Rutten GE, Ruis C, et al. Mild depressive symptoms do not influence cognitive functioning in patients with type 2 diabetes. Psychoneuroendocrinology. 2013;38(3):376–86. 22818834 10.1016/j.psyneuen.2012.06.014

[37] Ryan CM, van Duinkerken E, Rosano C. Neurocognitive consequences of diabetes. Am Psychol. 10.1037/a0040455 27690485

[38] Rawlings AM, Sharrett AR, Schneider AL, et al. Diabetes in midlife and cognitive change over 20 years: a cohort study. Ann Intern Med. 2014;161(11):785–93. 25437406 10.7326/M14-0737 PMC4432464

[39] van Eersel ME, Joosten H, Gansevoort RT, Dullaart RP, Slaets JP, Izaks GJ. The interaction of age and type 2 diabetes on executive function and memory in persons aged 35 years or older. PLoS ONE. 2013;8(12):e82991. Published 2013 Dec 18. 24367577 10.1371/journal.pone.0082991 PMC3867457

[40] Winkler A, Dlugaj M, Weimar C, et al. Association of diabetes mellitus and mild cognitive impairment in middle-aged men and women. J Alzheimers Dis. 2014;42(4):1269–77. 25024326 10.3233/JAD-140696

[41] Sola T, Pimiä E, Lahti E, Lahtela J, Jehkonen M. Type 2 diabetes and cognitive performance in middle age: a cross-sectional study. J Clin Exp Neuropsychol. 2023;45(4):423–32. 37642462 10.1080/13803395.2023.2246668

[42] Peng D, Geriatric Neurology Group, Chinese Society of Geriatrics; Clinical Practice Guideline for Cognitive Impairment of Cerebral Small Vessel Disease Writing Group. Clinical practice guideline for cognitive impairment of cerebral small vessel disease. Aging Med (Milton). 2019;2(2):64–73. Published 2019 Jun 20. 31942514 10.1002/agm2.12073 PMC6880706

[43] Rosenberg GA, Wallin A, Wardlaw JM, et al. Consensus statement for diagnosis of subcortical small vessel disease. J Cereb Blood Flow Metab. 2016;36(1):6–25. 26198175 10.1038/jcbfm.2015.172 PMC4758552

[44] Amiel SA, Dixon T, Mann R, Jameson K. Hypoglycaemia in type 2 diabetes. Diabet Med. 2008;25(3):245–54. 18215172 10.1111/j.1464-5491.2007.02341.x PMC2327221

[45] Deary IJ, Zammitt NN. Symptoms of hypoglycaemia and effects on mental performance and emotions. In: Frier BM, Heller S, McCrimmon RJ, editors. Hypoglycaemia in clinical diabetes. 3rd ed. Chichester, U.K.: Wiley-Blackwell; 2014. pp. 23–45.

[46] Aung PP, Strachan MW, Frier BM, et al. Severe hypoglycaemia and late-life cognitive ability in older people with type 2 diabetes: the Edinburgh type 2 diabetes study. Diabet Med. 2012;29(3):328–36. 22023662 10.1111/j.1464-5491.2011.03505.x

[47] Sommerfield AJ, Deary IJ, Frier BM. Acute hyperglycemia alters mood state and impairs cognitive performance in people with type 2 diabetes. Diabetes Care. 2004;27(10):2335–40. 15451897 10.2337/diacare.27.10.2335

[48] Stewart L, Moher D, Shekelle P. Why prospective registration of systematic reviews makes sense. Syst Rev. 2012;1:7. Published 2012 Feb 9. 22588008 10.1186/2046-4053-1-7 PMC3369816

[49] Stern C, Kleijnen J. Language bias in systematic reviews: you only get out what you put in. JBI Evid Synth. 2020;18(9):1818–9. 32925418 10.11124/JBIES-20-00361

[50] Sanderson S, Tatt ID, Higgins JP. Tools for assessing quality and susceptibility to bias in observational studies in epidemiology: a systematic review and annotated bibliography. Int J Epidemiol. 2007;36(3):666–76. 17470488 10.1093/ije/dym018

[51] Reijmer YD, van den Berg E, Ruis C, Kappelle LJ, Biessels GJ. Cognitive dysfunction in patients with type 2 diabetes. Diabetes Metab Res Rev. 2010;26(7):507–19. 20799243 10.1002/dmrr.1112

[52] Tomlin A, Sinclair A. The influence of cognition on self-management of type 2 diabetes in older people. Psychol Res Behav Manag. 2016;9:7–20. Published 2016 Jan 21. 26855601 10.2147/PRBM.S36238 PMC4727517

[53] American Diabetes Association. 12. Older adults: *Standards of Medical Care in Diabetes-2019*. Diabetes Care. 2019;42(Suppl 1):139–S147. 10.2337/dc19-S012 30559238

[54] Luchsinger JA, Lehtisalo J, Lindström J, et al. Cognition in the Finnish diabetes prevention study. Diabetes Res Clin Pract. 2015;108(3):e63–6. 25799080 10.1016/j.diabres.2015.02.023

[55] Zhao RR, O’Sullivan AJ, Fiatarone Singh MA. Exercise or physical activity and cognitive function in adults with type 2 diabetes, insulin resistance or impaired glucose tolerance: a systematic review. *Eur Rev Aging Phys Act*. 2018;15:1. Published 2018 Jan 22. 10.1186/s11556-018-0190-1 PMC5776769 29387262

[56] Biessels GJ, Strachan MW, Visseren FL, Kappelle LJ, Whitmer RA. Dementia and cognitive decline in type 2 diabetes and prediabetic stages: towards targeted interventions. Lancet Diabetes Endocrinol. 2014;2(3):246–55. 24622755 10.1016/S2213-8587(13)70088-3

[57] Domínguez RO, Marschoff ER, González SE, Repetto MG, Serra JA. Type 2 diabetes and/or its treatment leads to less cognitive impairment in Alzheimer’s disease patients. Diabetes Res Clin Pract. 2012;98(1):68–74. 22658669 10.1016/j.diabres.2012.05.013

[58] Exalto LG, Biessels GJ, Karter AJ, et al. Risk score for prediction of 10 year dementia risk in individuals with type 2 diabetes: a cohort study. Lancet Diabetes Endocrinol. 2013;1(3):183–90. 24622366 10.1016/S2213-8587(13)70048-2 PMC4429783

