# Supplementary material for: The Effects of Type 2 Diabetes on Cognitive Performance: A Review of Reviews
Source: Int J Behav Med. 2024 Mar 11;31(6):944–58. doi: 10.1007/s12529-024-10274-6 (PMC11588889; doi:10.1007/s12529-024-10274-6)
Supplement: Supplementary file 1 — Supplementary Material 1 [file 12529_2024_10274_MOESM1_ESM.docx]

**Table S1.** Results of the meta-analyses on attention

| Cognitive domain | Review | Tests analysed | k | n (gr 1 / gr 2) | SMD (95 % CI) |
| --- | --- | --- | --- | --- | --- |
| Attention (overall) | Kálcza-Jánosi 2013 | DSST, TMT-A, DVT | 4 | 136/136 | d = -0.33 (-0.56, -0.15)* |
|  | Monette 2014 | BSAT, BTA, CBT-F, DS-F, Stroop (pt. 3), TMT-B, WCST, M-WCST | 16 | 1440/7665 | d = -0.29 (-0.34, -0.24)* |
|  | Palta 2014 | DS-B, DS-F, Stroop (pt. 1, 2) | 14 | 2418/20725 | d = -0.19 (-0.26, -0.12)* |
|  | Pelimanni 2019 | DS-B, PASAT, STMT | 5 | 158/147 | g = -0.55 (-0.80, -0.30)* |
|  | Vincent 2015 | DSST, AM, PASAT, PST, WMS-A | 27 | 25 669 total | d = -0.38 (-0.48, -0.29)* |
| Attentional capacity | Palta 2014 | DS-F | 8 | 732/2941 | d = -0.18 (-0.27, -0.08)* |
|  | Pelimanni 2019 | DS-F | 2 | 71/64 | g = -0.65 (-1.20, -0.11)* |
|  | Sadanand 2016 | DS-F | 6 | NR | d = -0.13 (-0.23, -0.03)* |
|  | Vincent 2015 | DS-F | 17 | 5491 total | d = -0.17 (-0.27, -0.07)* |
| Complex attention | Mansur 2018 | DSST | 23 | 2608/13117 | g = -0.35 (-0.43, -0.27)* |
|  | Palta 2014 | DSST | 12 | 1059/765 | d = -0.33 (-0.45, -0.20)* |
|  | Pelimanni 2019 | DSST | 6 | 1925/11691 | g = -0.79 (-1.03, -0.55)* |
|  | Sadanand 2016 | DSST | 9 | 541/1178 | d = -0.22 (-0.37, -0.07)* |
|  | Vincent 2015 | DSST | 22 | 22414 total | d = -0.43 (-0.53, -0.33)* |
| Concentration | Monette 2014 | CBT-F, DS-F | 7 | 760/3486 | d = -0.15 (-0.21, -0.08)* |
|  | Monette 2014 | Stroop (pt. 3) | 5 | 271/1006 | d = -0.33 (-0.44, -0.22)* |
|  | Palta 2014 | Stroop (pt. 3) | 6 | 493/666 | d = -0.26 (-0.39, -0.12)* |
|  | Pelimanni 2019 | DVT | 2 | 74/67 | g = -0.55 (-0.89, -0.22)* |
|  | Pelimanni 2019 | Stroop (pt. 3) | 2 | 38/41 | g = -0.70 (-1.15, -0.25)* |
|  | Vincent 2015 | Stroop (pt. 3) | 11 | 4011 total | d = -0.32 (-0.44, -0.21)* |
| Divided attention | Mansur 2018 | TMT-B | 25 | 2369/17846 | g = -0.27 (-0.13, -0.41)* |
|  | Monette 2014 | BSAT, BTA, M-WCST, TMT-B, WCST | 13 | 1045/4546 | d = -0.36 (-0.42, -0.31)* |
|  | Palta 2014 | TMT-B | 10 | 586/569 | d = -0.39 (-0.52, -0.27)* |
|  | Pelimanni 2019 | TMT-B | 5 | 128/133 | g = -0.59 (-0.91, -0.26)* |
|  | Sadanand 2016 | TMT-B | 5 | 331/203 | d = -0.52 (-0.34, -0.70)* |
|  | Vincent 2015 | TMT-B | 16 | 16 545 total | d = -0.32 (-0.48, -0.17)* |

*Note* AM = Attention Matrix, BSAT = Brixton Spatial Anticipation Test, BTA = Brief Test of Attention, CBT-F = Corsi Block-Tapping Test Forward, DS = Digit Span (B = Backward, F = Forward), DSST = Digit Symbol Substitution Test, DVT = Digit Vigilance Test, gr 1 = patient group, gr 2 = control group, k = number of studies, M-WCST = Modified Wisconsin Card Sorting Test, n = number of participants, PASAT = Paced Auditory Serial Addition Test, PST = Perceptual Speed Task, STMT = Four-Word Short-Term Memory Test, TMT = Trail Making Test, WCST = Wisconsin Card Sorting Test, WMS-A: Wechsler Memory Scale Revised - Attention Index

* = statistically significant

**Table S2.** Results of the meta-analyses on construction and motor performance

| Cognitive domain | Review | Tests analysed | k | n (gr 1 / gr 2) | SMD (95 % CI) |
| --- | --- | --- | --- | --- | --- |
| Motor speed (overall) | Monette 2014 | FT, GPB, PPB | 4 | 360/2506 | d = -0.30 (-0.38, -0.23)* |
| Motor function (overall) | Palta 2014 | GPB, FT | 3 | 294/2080 | d = -0.36 (-0.52, -0.19)* |
| Motor function | Palta 2014 | GPB (dominant hand) | 2 | 115/96 | d = -0.60 (-0.90, -0.31)* |
|  | Palta 2014 | GPB (non-dominant hand) | 2 | 115/96 | d = -0.51 (-0.81, -0.22)* |

*Note* FT = Finger Tapping, GPB = Grooved Pegboard Test, gr 1 = patient group, gr 2 = control group, k = number of studies, n = number of participants, PPB = Purdue Begboard Test

* = statistically significant

**Table S3.** Results of the meta-analyses on executive functions

| Cognitive domain | Review | Tests analysed | k | n (gr 1 / gr 2) | SMD (95 % CI) |
| --- | --- | --- | --- | --- | --- |
| Executive functions (overall) | Kálcza-Jánosi 2013 | CT, D-KEFS, Stroop, TMT-B, WCST | 4 | 136/136 | d = -0.32 (-0.21, -0.46)* |
|  | Palta 2014 | Stroop (pt. 3), TMT-B, WCST | 12 | 680/1104 | d = -0.33 (-0.42, -0.24)* |
|  | Pelimanni 2019 | CT, VF (p, s), Stroop (pt. 3), TMT-B, WCST | 9 | 2001/12 173 | g = -0.51 (-0.69, -0.34)* |
|  | Vincent 2015 | AM, AS, BDT, BSAT, CBT (b, f), CTT (pt. 2), DIS (b, f), DS, DSST, Go/No-Go, HSCT, LNS, PASAT, PST, SST, Stroop (pt. 1, 2, 3), TMT-A, TMT-B, TMT-C, VF (p, s), VSWMT, WCST, WMS-A | 60 | 79 069 total | d = -0.25 (-0.30, -0.20)* |
| Inhibition | Vincent 2015 | Stroop (pt. 3), HSCT, Go/No-Go | 13 | 4131 total | d = -0.32 (-0.43, -0.21)* |
| Mental flexibility | Vincent 2015 | BSAT, TMT-B, TMT-C, WCST, CTT (pt. 2) | 22 | 24 243 total | d = -0.36 (-0.49, -0.24)* |
| Fluency (overall) | Monette 2014 | RFF, VF (p, s) | 17 | 1540/8547 | d = -0.26 (-0.30, -0.22)* |
|  | Vincent 2015 | VF (p, s) | 31 | 41 467 total | d = -0.22 (-0.28, -0.16)* |
| Phonemic fluency | Monette 2014 | VF (p) | 11 | 832/3098 | d = -0.28 (-0.34, -0.21)* |
|  | Pelimanni 2019 | VF (p) | 4 | 1861/12 032 | g = -0.39 (-0.85, 0.07) |
|  | Sadanand 2016 | VF (p) | 6 | 568/2207 | d = -0.35 (-0.53, -0.16)* |
|  | Vincent 2015 | VF (p) | 20 | 5427 total | d = -0.38 (-0.50, -0.26)* |
| Semantic fluency | Monette 2014 | VF (s) | 11 | 1122/7975 | d = -0.25 (-0.29, -0.21)* |
|  | Pelimanni 2019 | VF (s) | 2 | 45/47 | g = -0.59 (-1.25, 0.07) |
|  | Sadanand 2016 | VF (s) | 8 | NR | d = -0.15 (-0.20, -0.10)* |
|  | Vincent 2015 | VF (s) | 21 | 40 242 total | d = -0.16 (-0.21, -0.11)* |

*Note* AM = Attention Matrix, AS = Alpha Span, BDT = Baddeley Dual Task, BSAT = Brixton Spatial Anticipation Test, CBT = Corsi Block-Tapping Test (b = backward, f = forward), CT = Category Test, CTT = Colour Trails Test, DF = Design Fluency, DIS = Digit Symbol Test (b = backward, f = forward), D-KEFS = Delis-Kaplan Executive Function System, DS = Digit Span, DSST = Digit Symbol Substitution Test, gr 1 = patient group, gr 2 = control group, HSCT = Hayling Sentence Completion Test, k = number of studies, LNS = Letter-Number Sequencing, n = participants, PASAT = Paced Auditory Serial Addition Test, PST = Perceptual Speed Task, RFF = Ruff Figural Fluency, SST = Serial Subtraction Test, TMT = Trail Making Test, VF = Verbal Fluency (p = phonemic, s = semantic), VSWMT = Visuospatial Working Memory Task, WCST = Wisconsin Card Sorting Test, WMS-A: Wechsler Memory Scale Revised - Attention Index

* = statistically significant

**Table S4.** Results of the meta-analyses on intelligence/global cognition

| Cognitive domain | Review | Tests analysed | k | n (gr 1 / gr 2) | SMD (95 % CI) |
| --- | --- | --- | --- | --- | --- |
| Global cognition | Monette 2014 | All tests from their meta-analyses: tables S1, S2, S3, S5, S6, S7, S8, S9, S11 | 25 | 1908/10 132 | d = -0.25 (-0.29, -0.22)* |
| Intelligence | Kálcza-Jánosi 2013 | RPM, WAIS, WASI | 3 | 109/115 | d = -0.68 (-1.15, -0.21)* |
| Non-verbal reasoning | Monette 2014 | CT, MA, M-WCST (categories), PC, RPM (standard + advanced), TPT, WCST (categories) | 6 | 333/994 | d = -0.29 (-0.41, -0.17)* |

*Note* CT = Category Test, gr 1 = patient group, gr 2 = control group, k = number of studies, MA = Wechsler Adult Intelligence Scale: Matrix Reasoning, M-WCST = Modified Wisconsin Card Sorting Test, n = number of participants, PC = Wechsler Adult Intelligence Scale: Picture Completion TPT = Tactual Performance Test, RPM = Raven Progressive Matrices, WAIS = Wechsler Adult Intelligence Scale, WASI = Wechsler Abbreviated Scale of Intelligence

* = statistically significant

**Table** **S5.** Results of the meta-analyses on memory (overall)

| Cognitive domain | Review | Tests analysed | k | n (gr 1 / gr 2) | SMD (95 % CI) |
| --- | --- | --- | --- | --- | --- |
| Memory (overall) | Kálcza-Jánosi 2013 | CVLT, RAVLT, WMS-R, WMS-III | 4 | 141/141 | d = -0.50 (-0.82, 0.07) |
|  | Monette 2014 | All verbal + visual memory tests: see tables S6 and S7 | 20 | 1410/8056 | d = -0.20 (-0.28, -0.12)* |
| Immediate | Monette 2014 | Verbal + visual memory immediate recall trials: see Tables S6 and S7 ADAS-Cog #R (word list single learning trial), PLT #R (single learning trial), RAVLT #R | 18 | 1308/7882 | d = -0.21 (-0.25, -0,17)* |
| Delayed | Monette 2014 | Verbal + visual memory delayed recall trials: see Tables S6 and S7 RAVLT (trail A6) #R | 18 | 1326/7720 | d = -0.20 (-0.24, -0.15)* |

*Note* ADAS-Cog, CVLT = California Verbal Learning Test, PLT = Picture Learning Test, RAVLT = Rey Auditory Verbal Learning Test, gr 1 = patient group, gr 2 = control group, k = number of studies, n = number of participants, WMS-R = Wechsler Memory Scale Revised, WMS-III = Wechsler Memory Scale 3rd Edition, #R = number recalled

* = statistically significant

**Table S6.** Results of the meta-analyses on memory (verbal)

| Cognitive domain | Review | Tests analysed | k | n (gr 1 / gr 2) | SMD (95 % CI) |
| --- | --- | --- | --- | --- | --- |
| Verbal memory (overall) | Palta 2014 | CVLT, RAVLT, LM | 15 | 1349 / 3259 | d = -0.28 (-0.37, -0.19)* |
|  | Pelimanni 2019 | CVLT, DWRT, FCSRT, VEPA, LM | 4 | 1873/11661 | g = -0.39 (-0.44, -0.34)* |
| Immediate recall | Kálcza-Jánosi 2013 | CVLT, Guild Test, LM, NAI, VEPA | 5 | 182/188 | d = -1.08 (-2.21, -0.04)* |
| Delayed recall | Kálcza-Jánosi 2013 | CVLT, Guild Test, LM, NAI, VEPA | 6 | 215/221 | d = -0.79 (-1.81, 0.18) |
| Immediate: word-list | Mansur 2018 | RAVLT | 13 | 1240/1098 | g = -0.21 (-0.33, -0.09)* |
|  | Monette 2014 | CERAD-WL, HVLT, RAVLT, VEPA | 9 | 658/3046 | d = -0.27 (-0.34, -0.20)* |
|  | Palta 2014 | CVLT | 2 | 202/2036 | d = -0.40 (-0.53, -0.28)* |
|  | Palta 2014 | RAVLT | 7 | 891/396 | d = -0.19 (-0.49, 0.12) |
|  | Sadanand 2016 | RAVLT | 11 | 2108/21045 | d = -0.51 (-0.94, -0.09)* |
| Immediate: story | Monette 2014 | EBMT, EMT, Guild Test, RBMT, LM | 10 | 748/4184 | d = -0.18 (-0.23, -0.12)* |
|  | Palta 2014 | LM | 4 | 144/364 | d = -0.13 (-0.55, 0.30) |
|  | Pelimanni 2019 | LM | 2 | 74/67 | g = 0.04 (-0.65, 0.73) |
|  | Sadanand 2016 | LM | 4 | 289/913 | d = -0.24 (-0.52, 0.03) |
| Delayed: word-list | Monette 2014 | CERAD, HVLT, RAVLT, VEPA | 12 | 709/2968 | d = -0.22 (-0.28, -0.15)* |
|  | Palta 2014 | CVLT | 4 | 264/2487 | d = -0.33 (-0.47, -0.19)* |
|  | Palta 2014 | RAVLT | 6 | 535/335 | d = -0.27 (-0.45, -0.09)* |
|  | Sadanand 2016 | RAVLT | 12 | 2129/21064 | d = -0.47 (-0.90, -0.05)* |
| Delayed: story | Monette 2014 | EBMT, EMT, Guild Test, RBMT, LM | 10 | 748/4184 | d = -0.18 (-0.24, -0.12)* |
|  | Palta 2014 | LM | 4 | 144/364 | d = -0.18 (-0.66, 0.30) |
|  | Pelimanni 2019 | LM | 2 | 74/67 | g = -0.05 (-0.86, 0.75) |
|  | Sadanand 2016 | LM | 4 | 289/913 | d = -0.30 (-0.62, 0.01) |

*Note* CERAD-WL = Consortium to Establish a Registry for Alzheimer's Disease Word-list, CVLT = California Verbal Learning Test, DWRT = Delayed Word Recall Test, EBMT = East Boston Memory Test, EMT = Emotional Memory Test (Neutral Paragraph), FCSRT = Free and Cued Selective Reminding Test, gr 1 = patient group, gr 2 = control group, k = number of studies, LM = Wechsler Memory Scale: Logical Memory, n = number of participants, NAI = Nuernberg Alters Inventar, RAVLT = Rey Auditory Verbal Learning Test, RBMT = Rivermead Behavioral Memory Test, VEPA = Verbal Paired Associates

* = statistically significant

**Table S7.** Results of the meta-analyses on memory (visual)

| Cognitive domain | Review | Tests analysed | k | n (gr 1 / gr 2) | SMD (95 % CI) |
| --- | --- | --- | --- | --- | --- |
| Visual memory (overall) | Palta 2014 | ROCF, VR | 6 | 616/1138 | d = -0.26 (-0.38, -0.14)* |
|  | Pelimanni 2019 | ROCF, SDPALT | 3 | 88/91 | g = -0.15 (-0.38, 0.07) |
| Immediate | Kálcza-Jánosi 2013 | SDPALT, VIPA | 2 | 88/88 | d = -0.11 (-0.13, -0.10)* |
|  | Monette 2014 | BVRT, BVMT-R, LLT, SDPALT, VIPA, VR | 5 | 362/2259 | d = -0.21 (-0.29, -0.13)* |
|  | Palta 2014 | ROCF | 4 | 250/337 | d = -0.33 (-0.52, -0.15)* |
|  | Palta 2014 | VR | 2 | 208/882 | d = -0.18 (-0.55, 0.20) |
| Delayed | Kálcza-Jánosi 2013 | SDPALT, VIPA | 2 | 88/88 | d = -0.08 (-0.34, 0.32) |
|  | Monette 2014 | BVMT-R, PLT, SDPALT, TCF, VIPA, VR | 5 | 272/713 | d = -0.26 (-0.39, -0.14)* |
|  | Palta 2014 | ROCF | 4 | 408/256 | d = -0.38 (-0.54, -0.21)* |
|  | Palta 2014 | VR | 2 | 208/882 | d = -0.11 (-0.38, 0.15) |
|  | Pelimanni 2019 | ROCF | 2 | 38/41 | g = -0.63 (-1.07, -0.18)* |

*Note* BVMT-R = Brief Visuospatial Memory Test - Revised, BVRT = Benton Visual Retention Test, gr 1 = patient group, gr 2 = control group, k = number of studies, LLT = Location Learning Test, n = number of participants, PLT = Picture Learning Test, ROCF = Rey-Osterrieth Complex Figure Test, SDPALT = Symbol-Digit Paired-Associate Learning Test, TCF = Taylor Complex Figure Test, VIPA = Wechsler Memory Scale: Visual Paired-Associates, VR = Wechsler Memory Scale: Visual Reproduction

* = statistically significant

**Table S8.** Results of the meta-analyses on perception

| Cognitive domain | Review | Tests analysed | k | n (gr 1 / gr 2) | SMD (95 % CI) |
| --- | --- | --- | --- | --- | --- |
| Perception/construction (overall) | Monette 2014 | BD, CDT, CRT, EFT, JLO, OA, ROCF (copying), TCF (copying), TPT | 7 | 493/2957 | d = -0.18 (-0.25, -0.10)* |
|  | Pelimanni 2019 | BD, EFT, OA, ROCF (copying), TPT | 6 | 158/542 | g = -0.30 (-0.45, -0.14)* |
| Drawing | Pelimanni 2019 | ROCF (copying) | 3 | 70/71 | g = -0.60 (-0.94, -0.27)* |

*Note* BD = Wechsler Adult Intelligence Scale: Block Design, CDT = Clock Drawing Test, CRT = Card Rotations Test, EFT = Embedded Figures Test, gr 1 = patient group, gr 2 = control group, JLO = Judgement of Line Orientation, k = number of studies, n = number of participants, OA = Wechsler Adult Intelligence Scale: Object Assembly, ROCF = Rey-Osterrieth Complex Figure Test, TCF = Taylor Complex Figure Test, TPT = Tactual Performance Test

* = statistically significant

**Table S9.** Results of the meta-analyses on processing speed

| Cognitive domain | Review | Tests analysed | k | n (gr 1 / gr 2) | SMD (95 % CI) |
| --- | --- | --- | --- | --- | --- |
| Processing speed (overall) | Monette 2014 | EFT, C, CO, DSST, DVT, LDST, SDMT, SRT , TMT-A, NCT, OSDMT, SPCT, Stroop (pt. 1, 2) | 22 | 1678/7822 | d = -0.33 (-0.38, -0.29)* |
|  | Palta 2014 | TMT-A, DSST | 16 | 1381/1695 | d = -0.33 (-0.41, -0.26)* |
|  | Pelimanni 2019 | CRT, DSST, GPB, SDMT, SRT, TMT-A | 10 | 2063/11832 | g = -0.68 (-0.84, -0.52)* |
| Motor task demands | Monette 2014 | C, CO, DSST, DVT, LDST, SDMT, SRT , TMT-A | 21 | 1551/7125 | d = -0.37 (-0.41, -0.32)* |
| Oral task demands | Monette 2014 | NCT, OSDMT, SPCT, Stroop (pt. 1, 2) | 6 | 388/1001 | d = -0.14 (-0.24, -0.04)* |
| Color naming | Palta 2014 | Stroop (pt. 2) | 6 | 516/325 | d = -0.26 (-0.42, -0.10)* |
| Reaction time | Pelimanni 2019 | Choice Reaction Time | 2 | 63/66 | g = -0.51 (-0.85, -0.16)* |
|  | Pelimanni 2019 | Simple Reaction Time | 2 | 63/66 | g = -0.45 (-0.97, 0.08) |
| Reading | Palta 2014 | Stroop (pt. 1) | 6 | 516/325 | d = -0.28 (-0.45, -0.02)* |
| Visual search | Mansur 2018 | TMT-A | 22 | 1817/8754 | g = -0.31 (-0.15, -0.48)* |
|  | Palta 2014 | TMT-A | 11 | 811/1295 | d = -0.34 (-0.44, -0.24)* |
|  | Pelimanni 2019 | TMT-A | 3 | 53/58 | g = -0.50 (-1.26, 0.26) |
|  | Vincent 2015 | TMT-A | 13 | 5349 total | d = -0.47 (-0.60, -0.35)* |

*Note* C = Cancellation, CO = Wechsler Adult Intelligence Scale III: Coding, CRT = Choice Reaction Time, DSST = Digit Symbol Substitution Test, DVT = Digit Vigilance Test, EFT = Embedded Figures Test, GPB = Grooved Pegboard Test, gr 1 = patient group, gr 2 = control group, k = number of studies, LDST = Letter-Digit Substitution Test, n = participants, NCT = Number Comparison Test, OSDMT = Oral Symbol Digit Modalities Test, SDMT = Symbol Digit Modalities Test, SPCT = Salthouse Perceptual Comparison Test, SRT = Simple Reaction Time, TMT = Trail Making Test

* = statistically significant

**Table S10.** Results of the meta-analyses on verbal functions and language skills

| Cognitive domain | Review | Tests analysed | k | n (gr 1 / gr 2) | SMD (95 % CI) |
| --- | --- | --- | --- | --- | --- |
| Verbal functions (overall) | Kálcza-Jánosi 2013 | VCI (subtests not specified), VF | 2 | 68/68 | d = -0.36 (-0.45, -0.27)* |
|  | Pelimanni 2019 | BNT, SI, VO | 3 | 78/463 | g = -0.26 (-0.51, -0.02)* |

*Note* BNT = Boston Naming Test, gr 1 = patient group, gr 2 = control group, k = number of studies, n = number of participants, SI = Wechsler Adult Intelligence Scale: Similarities, VCI = Wechsler Adult Intelligence Scale: Verbal Comprehension Index, VF = Verbal Fluency, VO = Wechsler Adult Intelligence Scale: Vocabulary

* = statistically significant

**Table S11.** Results of the meta-analyses on working memory

| Cognitive domain | Review | Tests analysed | k | n (gr 1 / gr 2) | SMD (95 % CI) |
| --- | --- | --- | --- | --- | --- |
| Working memory (overall) | Kálcza-Jánosi 2013 | DS, LNS | 3 | 86/86 | d = 0.04 (-0.47, 0.54) |
|  | Monette 2014 | AS, CBT-B, DOT, STMT, LNS, DS-B | 11 | 1163/6582 | d = -0.20 (-0.24, -0.15)* |
|  | Pelimanni 2019 | DS-B, STMT, PASAT | 5 | 177/161 | g = -0.51 (-0.79, -0.22)* |
|  | Vincent 2015 | CBT-F/B, DIS-F/B, DS, LNS, AS, BDT, SST, VSWMT | 28 | 28 118 total | d = -0.13 (-0.19, -0.06)* |
| Reversing serial order | Palta 2014 | DS-B | 8 | 1938/20 070 | d = -0.12 (-0.22, -0.02)* |
|  | Pelimanni 2019 | DS-B | 3 | 95/81 | g = -0.66 (-1.38, 0.07) |
|  | Sadanand 2016 | DS-B | 9 | NR | d = -0.17 (-0.33, -0.02)* |
|  | Vincent 2015 | DS-B | 18 | 26 992 total | d = -0.24 (-0.35, -0.13)* |

*Note* AS = Alpha Span, BDT = Baddeley Dual Task, CBT = Corsi Block-Tapping Test (B = Backward, F = Forward), DOT = Digit Ordering Test, DIS = Digit Symbol (B = Backward, F = Forward), DS-B = Digit Span Backward, gr 1 = patient group, gr 2 = control group, k = number of studies, LNS = Letter-Number Sequencing, n = number of participants, NR = not reported, PASAT = Paced Auditory Serial Addition Test, SST = Serial Subtraction Test, STMT = Four-Word Short-Term Memory Test, VSWMT = Visuospatial Working Memory Task

* = statistically significant
